# Establishment of *Listeria monocytogenes* in the Gastrointestinal Tract

**DOI:** 10.3390/microorganisms7030075

**Published:** 2019-03-10

**Authors:** Morgan L. Davis, Steven C. Ricke, Janet R. Donaldson

**Affiliations:** 1 Center for Food Safety, Department of Food Science, University of Arkansas, Fayetteville, AR 72704, USA; mlw055@email.uark.edu (M.L.D.); sricke@uark.edu (S.C.R.); 2 Department of Cell and Molecular Biology, The University of Southern Mississippi, Hattiesburg, MS 39406, USA

**Keywords:** bile, Listeria, oxygen availability, pathogenic potential, gastrointestinal tract

## Abstract

*Listeria monocytogenes* is a Gram positive foodborne pathogen that can colonize the gastrointestinal tract of a number of hosts, including humans. These environments contain numerous stressors such as bile, low oxygen and acidic pH, which may impact the level of colonization and persistence of this organism within the GI tract. The ability of *L. monocytogenes* to establish infections and colonize the gastrointestinal tract is directly related to its ability to overcome these stressors, which is mediated by the efficient expression of several stress response mechanisms during its passage. This review will focus upon how and when this occurs and how this impacts the outcome of foodborne disease.

## 1. Introduction

Foodborne pathogens account for nearly 6.5 to 33 million illnesses and 9000 deaths each year in the United States [1]. There are over 40 pathogens that can cause foodborne disease. The six most common foodborne pathogens are *Salmonella*, *Campylobacter jejuni*, *Escherichia coli* O157:H7, *Listeria monocytogenes*, *Staphylococcus aureus*, and *Clostridium perfringens*. Together, these six pathogens are responsible for $2.9 to 6.7 billion in medical expenses in the United States each year [2]. Of these pathogens, *L. monocytogenes* possesses one of the highest mortality rates, suggesting a need for the understanding of its pathogenicity [3].

*L. monocytogenes* is a Gram positive, foodborne pathogen that was first identified in 1924 and later established as the causative agent of listeriosis in 1989 [4,5]. The severity of listeriosis can range from gastroenteritis to meningitis or encephalitis, septicemia or fetal infections [6]. Listeriosis has an approximate 20% mortality rate in the United States, where those most susceptible include the young, elderly, immunocompromised, and pregnant [3,7].

*L. monocytogenes* is found as a saprophyte in nature yet can tolerate the stressors encountered within the mammalian gastrointestinal tract, and also the food-processing environment [3,7]. According to the 2009 FDA Food Code, the food industry currently uses a combination of modified atmospheric and vacuum packaging, which helps to inhibit the growth of pathogens with a combination of oxygen, carbon dioxide and nitrogen. The inhibition of pathogenic organisms is accomplished by a higher concentration of carbon dioxide than oxygen within packaging after processing [8]. However, *L. monocytogenes* is able to overcome exposure to variations in oxygen concentrations that occur during processing and packaging of food.

*L. monocytogenes* can also tolerate the multiple stressors encountered within the gastrointestinal tract. These include bile salts, variations in pH, and available oxygen [9,10,11]. The initial stressor encountered following the consumption of food contaminated with *L. monocytogenes* is acidic conditions within the stomach, followed by bile within the intestines. Once in the gastrointestinal tract, *L. monocytogenes* has been shown to invade into the intestinal epithelial cells by the use of internalins A (InlA) and B (InlB) through Peyer’s patches [12,13], intestinal villi [14] and goblet cells [15]. This ability to invade allows *L. monocytogenes* to disperse through the body through the use of lymphoid cells [13]. After reaching the liver, *L. monocytogenes* is able to translocate into the gallbladder through bile canaliculi, where it can replicate extracellularly [16,17]. This allows for the recycling and re-entry of *L. monocytogenes* cells into the gastrointestinal tract through the biliary ducts [17]. Furthermore, *L. monocytogenes* has the capability of surviving and growing at temperatures ranging from 0.4 °C to 45 °C and pH ranging from 4.0 to 9.6 [18,19].

This review will describe the current literature as it relates to the genetics of *L. monocytogenes*, the bile induced stress response, and the impact of oxygen availability on survival in the gastrointestinal tract. The first section is a brief description of *L. monocytogenes* lineages and genomics. Because survival mechanisms and stress responses vary between strains, extensive genomic characterization of different serovars has been done in order to understand pathogenesis. The second section of this review will explore the antimicrobial conditions encountered within the mammalian host, specifically the high concentration of bile salts in the gastrointestinal tract and defense mechanisms that assist in the survival of *L. monocytogenes* against bile. The final section will focus on oxygen availability as an environmental factor that influences the regulation of stress response genes and virulence factors.

## 2. Serovars of Listeria monocytogenes

There are four genetic lineages and thirteen serovars of *L. monocytogenes* [20,21]. The majority of *L. monocytogenes* isolates are part of the lineages I and II and were first characterized in 1989 [22]. Two additional lineages, III and IV, have subsequently been identified in 1995 and 2008, respectively [23,24]. Lineage I consists of serovars 1/2b, 3b, 3c, and 4b. Lineage II consists of serovars 1/2a, 1/2c, and 3a. Serovars 4a, 4b, and 4c comprise lineages III and IV. Serovars 1/2a, 1/2b, 1/2c and 4b make up roughly 90% of human listeriosis cases [25]. Serovar 4b is responsible for most foodborne outbreaks and sporadic cases [26]. Therefore, these serovars may possess specific virulence factors that improve the capability of particular strains to cause systemic infections, such as the ability to invade intestinal epithelial cells [27]. 

The genome content of 113 strains has been analyzed using DNA macroarray hybridizations. Only the presence or absence of genes were observed. Results indicated that many of the genomic differences between these strains were associated with surface proteins and genes involved in sugar metabolism [28]. These could serve as genes necessary for adaptation to various environments, including virulence factors required for intracellular replication [29]. Genomic analysis of serovar 4b revealed a group of surface protein genes that are specific to *L. monocytogenes.* These include *inlA*, *inlB*, *inlC*, *inlG*, *inlH*, *inlE*, and *inlF* [28]. Interestingly, analysis of serovar 4a, which is mostly associated with animal cases of listeriosis, revealed a lack of internalin genes aside from *inlAB* thus reflecting their lower potential to cause disease in humans [28].

Proteins involved in the phosphotransferase system (PTS) of glucose have been shown to influence the expression of virulence genes [30]. PrfA is the major regulator of virulence in *L. monocytogenes*, where it controls the expression of multiple genes including those encoding listeriolysin O and ActA, both of which are involved in cell-to-cell dissemination and invasion [31,32]. Through the decrease in expression of PTS operons and transport of sugars when PrfA is overexpressed, it is possible that PrfA activity is controlled by a direct interaction with the PTS [33]. PTS (mannose) contributes to the down regulation of virulence gene transcription in glucose medium [34]. However, a genetic analysis of several strains indicated that β-glucoside-specific PTS was not found in lineage II, which is made up of serovars 4b, 4d, 4e, 7, 1/2b, and 3b [28]. Therefore, another PTS system must be utilized in the regulation of the PrfA regulon in these serovars of lineage II. Specifically, serovar 4b may have evolved other PTSs that are more efficient within a mammalian host due to its prevalence in hospital cases and deaths.

A recent genomic comparison between serovars 1/2a demonstrated the conservation of multiple genes associated with virulence including internalins, PrfA, and various sRNAs [35]. Mutations were found in PrfA in EGD-e compared to EGD [35]. This indicates that genomic analyses alone cannot determine pathogenic potential. Sigma B, an alternative stress factor controlled by PrfA, has been shown to contribute to virulence [36]. An overexpression of both PrfA and sigma B have been observed in lineage II serovars [37,38]. This could be associated with the fact that these are isolates known to inhabit and adapt to multiple environments (inside or outside a host). Though sigma B was identified in lineage I and III but not IV, expression was decreased upon exposure to acidic (pH 2.5) and oxidative stress. Another recent study compared the genomes of 4b strains to determine links related to outbreaks of *L. monocytogenes* [39]. This study identified three clades among the 4b strains tested, 28 SNPs were found that were unique to 4b variant strains of that were not present in lineage II among the lmo037-lmo037 region. Genomic comparisons between these strains indicated that the majority of genes unique to the 4bV strains included primarily hypothetical and phage related genes. Seven were found to be internalin or internalin-like genes and others may be involved in survival in unique environments, such as ferrous iron transport. In addition to relatedness among clades, another study indicated relatedness of 4bV strains based on geographic location [40].

Recently, a genome-scale metabolic model was completed against 6 different strains of *L. monocytogenes* [41]. There were 1,116 metabolites contained in the 6 strains. Unique metabolites were found in strain J2-064 (p-Hydroxybenzaldehyde) and serovar 1/2a strains tested (Toxopyrimidine). There were also differences in the nutrients utilized between the strains tested that indicated serovar specific differences. 

Another recent study identified that 8 genes were highly significantly associated with food isolates from lineage II [42]. In this study, 174 isolates were compared to identify genes and SNPs that were associated with food or clinical isolates. The 8 genes identified were mostly hypothetical genes. It is necessary to further characterize these genes to determine the role they have in *L. monocytogenes* survival. Additionally, of the isolates analyzed, 45 out of 125 stress related genes were either missing or truncated in some isolates. There are also differences in the presence of internalization genes. For instance, lmo1082 occurs more frequently in lineage II than lineage I, yet lmo0320 was found more frequently among lineage I than lineage II. This indicates a necessity for characterizing isolates from various serovars based on their infection ability as a means to determine similarities among serovars.

There are both technical and functional limitations in analyzing the genome for pathogenic potential. Possession of genes related to virulence may not necessarily correlate with pathogenic potential. In order to determine the function of a gene product, it is necessary to examine expression under various conditions. One must also consider the presence of hot spots (regions with elevated recombination rates) within the genome that can continually alter the gene sequences.

## 3. Acidity Induced Stress Response

Upon ingestion, *L. monocytogenes* encounters the stressor of acidic conditions within the stomach as a first barrier to bacterial invasion. Several mechanisms have been acquired in order to overcome this environment, including the acid tolerance response (ATR) and the glutamate decarboxylase system (GAD). According to O’Driscoll and colleagues (1996), survival assays showed that a pre-exposure to a pH of 5.5 increased the resistance of *L. monocytogenes* to a pH of 3.5. This suggests that the ATR assists in tolerance of acidic conditions after adaptation [43]. The ATR two-component pathway also provides cross protection of *L. monocytogenes* against high temperatures, oxidative and osmotic stress [43]. Through this protection, survival following exposure to stressors found throughout the gastrointestinal tract is increased and thus may be essential to the ultimate development of listeriosis.

The GAD system has been thoroughly characterized and found to assist in acid tolerance. Upon exposure to low pH, glutamate is converted to γ-aminobutyrate (GABA) [44]. This reaction occurs through the consumption of protons which reduces the proton concentration in the cell resulting in lower pH [44]. Not all *L. monocytogenes* strains possess the GAD system; it has been shown to be required by certain strains for maintaining homeostasis within gastric juices [45]. The expression of GAD is induced upon exposure to chloride ions, low pH and anaerobiosis [46,47,48]. Activation of GAD is stimulated within the stomach upon exposure to low pH but provides cross protection against anaerobiosis and bile salts within the small intestine and potentially the gall bladder. However, recent studies have shown that acid shock at low temperatures of 25 °C may induce *prfA* [49]. This suggests that low pH could serve as a trigger for the expression of virulence and stress response.

## 4. Bile Induced Stress Response

Bile is produced in the liver, stored in the gall bladder, and released into the small intestine upon ingestion of food; bile is composed of pigments, cholesterol, bile salts, and water. Bile salts are saturated, hydroxylated C-24 cyclopentanophenanthrene sterols that are synthesized in the hepatocytes from cholesterol as chenodeoxycholic acid and cholic acid [50].

Bile is released from the liver through the left and right hepatic ducts forming the common duct, which connects to the gall bladder. Once in the gall bladder, the bile is concentrated by as much as 5 to 10-fold through the removal of water and electrolytes [51,52]. Bile salts are further metabolized by conjugation to glycine or taurine, which decreases their p*K*_a_ value to about 5 [50]. After a meal, bile from the gall bladder is released into the common duct where it enters the duodenum of the small intestine [52]. Once bile is secreted into the intestinal tract, a majority of bile salts are reabsorbed and recycled through the enterohepatic circulation [53].

Bile salts are amphipathic molecules that have been shown to possess antimicrobial properties; bile salts have been shown to degrade viral and bacterial membranes containing lipids and also induce DNA damage [54,55,56]. In order for enteric pathogens to survive, they must possess mechanisms to resist these antimicrobial effects of bile salts. *L. monocytogenes* possesses numerous mechanisms to allow for resistance against bile, including the bile salt hydrolase *bsh* [57,58], the general stress response sigma factor *sigB* [51,59], the bile exclusion system *bilE* [60], and virulence regulator *prfA* [58]. The bile salt hydrolase catalyzes the deconjugation of bile acids and is produced by several Gram-negative and Gram-positive enteric bacteria such as *Enterococcus* [61] and *Lactobacillus* [62]. This deconjugation occurs through the hydrolysis of an amide bond that links bile acids to their amino acid conjugate glycine or taurine [63]. Studies have also shown that deletion of the *bsh* gene reduces the ability of *L. monocytogenes* to cause systemic infections by reducing the colonization potential within the gastrointestinal tract [57,58]. This demonstrates the importance of the bile salt hydrolase as one of the first defenses used to overcome stressors encountered within the gastrointestinal tract [57,58].

The general stress response sigma factor *sigB* is involved in regulating the expression of osmolyte transporters, such as OpuC, and is also involved in regulating processes needed for survival during oxidative stress, reduced pH, and starvation. The *sigB* transcription factor also serves as a positive regulator of factor A (PrfA), thus leading to the activation and regulation of major virulence factors [64]. Additionally, studies have shown a connection between *sigB* and the expression of genes related to bile resistance such as *bilE* and *bsh* [60,65]. BilE, the bile exclusion system, serves to prevent bile from entering the cell. It is also LexA regulated [66], indicating an association of bile resistance with the SOS response. LexA is auto-catalytically cleaved after interacting with RecA, which disrupts the DNA-binding portion of LexA resulting in DNA repair. PrfA serves as the virulence regulator and overlap has been identified between the *prfA* response and activation of stress response mechanisms similar to SigB [67,68].

Since bile is toxic to most pathogens, the gall bladder is usually a sterile environment. However, *L. monocytogenes* is able to overcome the toxicity of this environment and grow extracellularly. It has been proposed that extracellular growth within the gall bladder may provide an efficient route of transmission where the transit from the bile duct to the intestines could occur in as little as five minutes [17]. Expression of internalins were found to increase in avirulent strain HCC23 (internalin A) but decrease in virulent strains [69]. This suggests that bile differentially regulates the invasive potential of *L. monocytogenes*.

## 5. Anaerobiosis Induced Stress Response

Oxygen availability varies throughout the gastrointestinal tract. In the gastrointestinal tract, the stomach is microaerophilic (1 to 3% oxygen) and becomes more anaerobic within the intestines [70]. Carbon dioxide is known to inhibit the growth of most bacteria [71] and is found as a byproduct of reactions with acid in the stomach. The amount of carbon dioxide produced varies from individual to individual. Jydegaard-Axelson and colleagues [8] observed an increase in the expression of genes needed for survival under acidic conditions and an increase in branch-chain fatty acids in the cell membrane when *L. monocytogenes* is cultured under elevated carbon dioxide and anaerobic conditions. In addition, gene expression changed for invasion-associated internalin proteins InlA and LmaA, which are involved in attachment and invasion of host mammalian cells. Therefore, the increase in expression of invasion proteins could be attributed to a preference to escape the acidic environment.

It has previously been concluded that atmospheric environmental factors could contribute to the ability of *L. monocytogenes* to survive in the presence of stressors [72]. Studies have demonstrated that the activity of the bile salt hydrolase increases under anaerobic conditions [58]. Buchanan and Klawitter [67] observed that anaerobic conditions increased survival of *L. monocytogenes* under acidic conditions (pH 4.5). Also, oxygen restriction enhances growth at lower temperatures (approximately 19 °C). Together, these data suggest that oxygen availability could influence bile resistance and therefore the virulence capability of *L. monocytogenes.*

*L. monocytogenes* is a facultative anaerobe, which allows the bacterium to be able to undergo aerobic respiration, fermentation, and anaerobic respiration. However, this is still dependent upon the ability to respond to oxygen availability. This environmental sensing is typically controlled by a two-component signal transduction system which consists of a membrane bound sensor and a cytoplasmic response regulator [73]. Although little research has been conducted to analyze the connection of anaerobiosis to increased survival in the presence of stressors in *L. monocytogenes*, much is known about other Gram-positive organisms. In various Gram-positive bacteria, such as *Staphylococcus aureus*, *Bacillus subtilis*, and *Mycobacterium tuberculosis*, two-component systems have been shown to regulate metabolism and the expression of virulence factors in response to decreased oxygen concentrations [74,75,76]. For instance, the SrrAB two-component system of *S. aureus* is involved in the activation of stress response proteins, specifically those involved in DNA repair, the oxidative stress response and the alternative sigma factor, SigB, in oxygen limited environments [77]. However, this activation is in conjunction with multiple two-component systems [78].

The two-component system ResDE of *B. subtilis*, homologous to SrrAB in *S. aureus*, has been shown to regulate virulence factors, sporulation, and fermentation in *B. subtilis* [75,76]. A homolog to *resD* has been characterized in *L. monocytogenes.* ResD was found to influence the activity of *prfA* in *L. monocytogenes,* which in turn alters the expression of several virulence genes, including *inlA* [79]. This suggests that ResD may be an important factor in the regulation of virulence factors and stress responses under low-oxygen conditions.

In *M. tuberculosis*, DosSR (DevSR) is a two-component system that consists of DosS histidine kinase and DosR response regulator. The DosSR system is induced under various levels of stress such as carbon starvation and heat shock [80]. DosSR is also induced by hypoxia, thus indicating that this is an oxygen sensor [81]. A recent genomic study identified DosP in *L. monocytogenes,* which is similar to the histidine kinase found in *M. tuberculosis,* suggesting that *L. monocytogenes* belong to the category of Gram-positives that possess an oxygen sensor [82,83]. Similarity between Gram-positive organisms suggests a link between virulence, stress response and two-component signal transduction systems that affect the organisms’ ability to detect oxygen levels.

A recent study by Wright et al. [78] has identified a potential link between oxygen availability and bile resistance by comparing growth of several strains of *L. monocytogenes* in 0%, 1%, 5%, and 10% porcine bile. Results indicated that bile resistance increased under anaerobic conditions compared to aerobic for virulent strains F2365, 10403S and EGD-e but not avirulent strain HCC23. A comprehensive total proteomic study to identify mechanisms (metabolism and stress response) found that proteins associated with the cell envelope, membrane bioenergetics, cell division, and dehydrogenases involved in NADH:NAD+ alteration were increased under anaerobic conditions [84]. This suggests that these proteins may be involved in bile resistance during growth anaerobically. However, an oxygen sensor that may regulate these mechanisms has not been identified.

## 6. Conclusions

Numerous response systems have been found that confer protection against multiple stressors, including pH, bile, osmolarity and temperature. However, the influence of reduced oxygen availability has not been thoroughly characterized in these systems. For instance, several studies that have analyzed and characterized the bile resistance mechanisms of *L. monocytogenes* were conducted under aerobic conditions. The conditions of the gallbladder and small intestine, where bile salt concentrations are at its highest, is an environment ranging from microaerophilic to anaerobic [85]. Therefore, aerobic conditions do not accurately model physiological conditions within the human gastrointestinal tract.

Recent research has begun to suggest a connection between oxygen availability and the stress response in Gram-positive bacteria. Future studies include the identification of the potential oxygen sensor responsible for detecting available oxygen concentrations and development of drugs to target this sensor to reduce and prevent infection with *L. monocytogenes*. Therefore, it is imperative that the oxygen response of *L. monocytogenes* be characterized relative to the stress response.
